# Multiparticulate Systems of Ezetimibe Micellar System and Atorvastatin Solid Dispersion Efficacy of Low-Dose Ezetimibe/Atorvastatin on High-Fat Diet-Induced Hyperlipidemia and Hepatic Steatosis in Diabetic Rats

**DOI:** 10.3390/pharmaceutics13030421

**Published:** 2021-03-20

**Authors:** Carlos Torrado-Salmerón, Víctor Guarnizo-Herrero, Joana Henriques, Raquel Seiça, Cristina M. Sena, Santiago Torrado-Santiago

**Affiliations:** 1 Department of Pharmaceutics and Food Technology, Faculty of Pharmacy, Complutense University of Madrid, Plaza Ramón y Cajal s/n, 28040 Madrid, Spain; ctorrado@ucm.es (C.T.-S.); victor08@ucm.es (V.G.-H.); 2 Institute of Physiology, Faculty of Medicine, University of Coimbra, 3000‐548 Coimbra, Portugal; joanafhenriques@gmail.com (J.H.); rmfseica@gmail.com (R.S.); csena@ci.uc.pt (C.M.S.); 3 Institute for Clinical and Biomedical Research (iCBR), Faculty of Medicine, University of Coimbra, Azinhaga de Santa Comba, Celas, 3000-548 Coimbra, Portugal; 4 Instituto Universitario de Farmacia Industrial (IUFI), Complutense University of Madrid, Plaza Ramón y Cajal s/n, 28040 Madrid, Spain

**Keywords:** ezetimibe, atorvastatin, solid dispersion, micellar system, hyperlipidemia, liver steatosis

## Abstract

The aim of this study was to develop multiparticulate systems with a combination of ezetimibe micellar systems and atorvastatin solid dispersions using croscarmellose as a hydrophilic vehicle and Kolliphor RH40 as a surfactant. The presence of a surfactant with low hydrophilic polymer ratios produces the rapid dissolution of ezetimibe through a drug–polymer interaction that reduces its crystallinity. The solid dispersion of atorvastatin with low proportions of croscarmellose showed drug–polymer interactions sufficient to produce the fast dissolution of atorvastatin. Efficacy studies were performed in diabetic Goto-Kakizaki rats with induced hyperlipidemia. The administration of multiparticulate systems of ezetimibe and atorvastatin at low (2 and 6.7 mg/kg) and high (3 and 10 mg/kg) doses showed similar improvements in levels of cholesterol, triglycerides, lipoproteins, alanine transaminase, and aspartate transaminase compared to the high-fat diet group. Multiparticulate systems at low doses (2 and 6.7 mg/kg of ezetimibe and atorvastatin) had a similar improvement in hepatic steatosis compared to the administration of ezetimibe and atorvastatin raw materials at high doses (3 and 10 mg/kg). These results confirm the effectiveness of solid dispersions with low doses of ezetimibe and atorvastatin to reduce high lipid levels and hepatic steatosis in diabetic rats fed a high-fat diet.

## 1. Introduction

Diabetes is a progressive metabolic disorder that increases the prevalence of nonalcoholic fatty liver disease (NAFLD). This hepatic damage is manifested in liver steatosis and inflammation of the hepatic cells. Non-alcoholic steatohepatitis (NASH) is also characterized by a high level of cholesterol (TC), triglycerides (TG), and low-density lipoprotein (LDL) in individuals with hyperlipidemia [1]. The accumulation of LDL and high values of transaminases such as alanine transaminase (ALT) and aspartate transaminase (AST) are indicative of significant hepatic steatosis in diabetic patients and are difficult to manage with current available therapies [1]. Animal models, such as Goto-Kakizaki (GK) rats are especially useful as models of diabetic dyslipidemia for the efficacy studies of different pharmacological treatments [2].

Ezetimibe (EZ) and atorvastatin calcium (ATV) are hypolipidemic agents that are administered in association. Both active pharmaceutical ingredients (APIs) are classified as class II compounds (poorly aqueous solubility) in the biopharmaceutical classification system (BCS). The low oral bioavailability of EZ and ATV were mainly attributed to their poor aqueous solubility [3,4], although in recent years, various approaches such as solid dispersion (SD) and micellar system (MS) have been used to improve their solubility and bioavailability [4,5]. However, recent studies have shown that concentrations in the liver are more important than high plasma bioavailability for the treatment of hepatic steatosis in hyperlipidemic patients [6]. To improve the poor solubility of EZ, surfactants such as Kolliphor RH40 have been used in micellar systems [7], while hydrophilic cellulose polymers such as Hydroxypropyl cellulose (HPC) or sodium croscarmellose have been selected for their pronounced hydrophilic properties [8,9] for the elaboration of solid dispersions of ATV, although EZ and ATV are difficult to quantify due to their low concentrations in liver tissue, meaning that the hepatic biodistribution of these active ingredients cannot be analyzed. Recent studies on other hydrophobic drugs such as voriconazole and amphotericin B showed a high liver biodistribution with similar micellar systems [10]. These active ingredients have shown an enterohepatic circulation, and the changes in the enterohepatic circulation of these drugs could be analyzed with an efficacy study in vivo in order to compare the differences between the formulations administered [11].

The use of a hepatic steatosis rat model can be effective in evaluating the efficacy in the liver of different hydrophilic systems [3,6]. Solid dispersions and micellar systems with surfactants delayed the metabolism of ezetimibe and atorvastatin and produced significant changes in NASH disorders [12,13]. These improvements in NASH levels may be particularly important in reducing the doses of EZ and ATV, and thus mitigating their adverse effects [6].

The aim of this study was to develop micellar systems of EZ with Kolliphor RH40 (MS-EZ) and solid dispersions of ATV (SD-ATV) with croscarmellose to investigate the improvement in the solubility and dissolution rate of these drugs. In this article, multiparticulate systems (MPS) were prepared with combinations of MS-EZ and SD-ATV in order to investigate whether these hydrophilic systems decrease hepatic steatosis in a diabetic dyslipidemia rat model.

## 2. Materials and Methods

### 2.1. Materials

Ezetimibe (EZ) and atorvastatin calcium (ATV) were obtained from Normon Pharmaceutical Co., Ltd. (Madrid, Spain). Croscarmellose sodium (Ac-Di-Sol SD-711) was purchased from FMC Corporation (Philadelphia, PA, USA). Kolliphor RH40 was purchased from Basf Chemical Company (Barcelona, Spain). Water was obtained from a Milli-Q water purification system (Billerica, MA, USA). All reagents and chemicals used were of analytical grade.

### 2.2. Methods

#### 2.2.1. Preparation of Formulations

EZ and ATV raw materials (EZ-RM and ATV-RM) were used as references in the “in vitro” and “in vivo” studies. The physical mixture of EZ (PM-EZ 1:2.5) was formulated by mixing 100 mg of EZ and 250 mg of croscarmellose sodium. The physical mixture of ATV (PM-ATV 1:1) was prepared using the same process but mixing 100 mg of ATV and 100 mg of croscarmellose sodium.

The solid dispersion of ezetimibe (SD-EZ) were prepared by dissolving 100 mg of EZ in 500 μL of ethanol, dissolved by the vortex (Fisherbrand TM; Milan, Italy) at 2500 rpm for 2 min. The dissolution of EZ was mixed in a ceramic bowl with 250 mg of croscarmellose for the SD-EZ (1:2.5) and 500 mg of croscarmellose for the SD-EZ (1:5), then dried using a drying oven (Memmert UN30, Schwabach, Germany) at 40 °C for 24 h. The solid dispersions were sieved to isolate the 0.297–0.850 mm fraction.

For the preparation of the micellar systems of ezetimibe (MS-EZ), 100 mg of EZ was dissolved in 500 μL of ethanol with 25 mg and 100 mg of Kolliphor RH40 for MS-EZ (1:0.25) and MS-EZ (1:1), respectively. Each solution was mixed with 250 mg of sodium croscarmellose and then dried at 40 °C for 24 h. The final product was screened to isolate the 0.297–0.840 mm fraction. 

The solid dispersion of atorvastatin (SD-ATV) was made following a similar process. A solution of 100 mg of ATV in 500 μL of ethanol was mixed with 100 and 300 mg of croscarmellose sodium for SD-ATV (1:1) and SD-ATV (1:3), respectively. The solid dispersions were dried at 40 °C for 24 h. The final product was screened to isolate the 0.297–0.840 mm fraction. 

Finally, the multiparticulate system formulations of EZ and ATV (MPS-I and MPS-II) were prepared with MS-EZ (1:1) and SD-ATV (1:1) formulations. MPS-I was prepared by mixing 380 mg of MS-EZ (1:1) and 583 mg of SD-ATV (1:1), and MPS-II by adding 253 mg of MS-EZ (1:1) and 390 mg of SD-ATV (1:1).

#### 2.2.2. Scanning Electron Microscopy (SEM)

Samples were mounted and sputtered under vacuum with a thin gold–palladium layer using a sputter coater metallizator Q150RS (Quorum technologies, Laughton, UK). After coating, the samples were analyzed with a Jeol JSM-6400 (Jeol Ltd., Peabody, MA, USA) scanning electron microscope with an acceleration voltage of 20 kV. All micrographs were analyzed by secondary electron imaging using an energy dispersive X-ray (EDX) technique for surface morphology, identifying the molecular composition of the EZ and ATV formulation particles at a magnification of 3000×. 

#### 2.2.3. Differential Scanning Calorimetry (DSC)

Samples were mounted on a TC 15 thermal analyzer (Mettler Toledo, Schwerzen-bach, Switzerland). The temperature was calibrated using the indium reference standard. Samples were accurately weighed into aluminum pans, then hermetically sealed with aluminum lids and heated from 25 °C to 250 °C at a heating rate of 10 °C/min under constant purging of dry nitrogen at 20 mL/min. Assuming a proportional relationship between crystallinity and fusion enthalpy, we estimated 100% crystallinity from the fusion enthalpy for EZ, ATV, and croscarmellose sodium raw materials [14,15]. An empty pan sealed in the same way as the samples was used as a reference.

#### 2.2.4. Powder X-ray Diffraction (PXRD)

The PXRD patterns were recorded on a Philips X’Pert-MPD X-ray diffractometer (Malvern Panalytical, Almelo, the Netherlands), in the CAI XRD (UCM, Madrid, Spain). The samples were irradiated with monochromatized CuKα radiation (λ = 1.542 Å) and analyzed between the 5 and 40° (2θ) degree range, scanning at a step size of 0.04° and a time of 1 s per step in all cases. The voltage and current used were 30 kV and 30 mA, respectively.

The degree of crystallinity (Xc) of the EZ for the different systems was determined semi-quantitatively via the mean of the decrease of the total area of the curve of the mean crystallinity of the 3 characteristic peaks at 16.44°, 19.13°, and 20.37° 2θ [14].

#### 2.2.5. Dissolution Studies

##### Sink Conditions

Dissolution studies under sink conditions were performed using the United States Pharmacopeia (USP) paddle method at 37.0 ± 0.5 °C (apparatus 2) in Erweka DT 80 (Erweka GmbH; Langen, Germany). Amounts equivalent to 10 mg of EZ or ATV were used in the dissolution vessels. 

The ezetimibe dissolution study was performed at 50 rpm with 500 mL of dissolution medium containing 0.45% of sodium lauryl sulphate in 0.05 M of sodium acetate buffer, adjusted to pH 4.5. The use of a medium with sodium lauryl sulphate is necessary to increase the low solubility of the active ingredient, this dissolution test method of ezetimibe is established by the United States Pharmacopeia (USP42-NF37, 2019). At different times, the samples were filtered by 0.45 μm (Acrodisc, NY, USA). The quantity of EZ was determined at 233 nm using a UV–VIS Jasco V-730 spectrophotometer (Tokyo, Japan) by the following calibration curve y = 0.0394x (μg/mL) − 0.0089 (r^2^ = 0.9996) across a range of 1–15 μg/mL. 

The atorvastatin dissolution study was performed at 75 rpm with 900 mL of dissolution medium containing 0.05 M phosphate buffer, adjusted to pH 6.8. This dissolution test method of atorvastatin is established by the United States Pharmacopeia (USP42-NF37, 2019). Samples were collected periodically and filtered through 0.45 μm; ATV was then determined at 241 nm by the following calibration curve: y = 0.0404x (μg/mL) − 0.0165 (r^2^ = 0.9994) with a range of 2–20 μg/mL. Both dissolution methods were validated according to ICH Q2 (R1) (CPMP/ICH/381/95). Each determination was performed in triplicate and the error bars on the graphs represent the standard deviation.

##### Non-Sink Conditions in Biorelevant Media

Ezetimibe and atorvastatin were evaluated in a biorelevant media under non-sink conditions. The dissolution study of the different formulations was performed at 100 rpm and 37.0 ± 0.5 °C using a magnetic stirrer thermostatic bath (FisherbrandTM; Milan, Italy). Amounts equivalent to 50 mg of EZ and ATV were added in 50 mL of pH 6.5 FaSSIF (fasted-state simulated intestinal fluid) biorelevant media purchased from biorelevant.com (London, UK). At different times, 0.5 mL samples were withdrawn and filtered through 0.45 μm (Acrodisc, NY, USA), and 0.3 mL was discarded before collecting the final 0.2 mL of filtrate. The filtered samples were immediately diluted in methanol and analyzed [16,17].

The sample quantification was carried out by HPLC (Agilent 1100 series FLD G1321A) equipped with a 50 µL loop. Samples were separated using a Zorbax SB C-8 column (4.6 × 250 mm, 5 µm). The mobile phase consisted of acetate buffer pH 4.0 (40%) and HPLC-grade acetonitrile (60%), with a flow rate of 1 mL/min. Ezetimibe was analyzed with a UV–VIS detector set at a wavelength of 233 nm and atorvastatin at 241 nm.

#### 2.2.6. Animal Study

This animal study was carried out in the Animal Experimentation Center in the faculty of medicine at the University of Coimbra under the guidelines of the Ethical Committee (Directive 2010/63/EU), with the project identification code 25/2015 (25 September 2015). Thirty-one male diabetic Goto-Kakizaki (GK) rats weighting between 350 and 400 g were used in the study. The animals were divided into 5 different groups (*n* = 6): 1 control group with a normal diet (Diet AO3-SAFE, Auxerre, France), 1 group fed a high-fat diet (HFD), and 3 treatment groups (EZ/ATV-RM, MPS-I, and MPS-II) fed a high-fat diet. The high-fat diet (SAFE, Auxerre, France), enriched with 7.5% cocoa butter and 1.25% cholesterol, was administered 4 weeks before treatment and 8 weeks during the treatment.

The formulations were suspended in 0.4 mL of an aqueous solution of sodium carboxymethyl cellulose (0.75% *w*/*v*) and administered by oral gavage after the suspension preparation to the treatment groups for 8 weeks. The doses of EZ/ATV-RM and MPS-I, added to the sodium carboxymethyl cellulose solution, were equivalent to 3 mg/kg of EZ and 10 mg/kg of ATV, and the doses of MPS-II were equivalent to 2 mg/kg of EZ and 6.7 mg/kg of ATV. At the end of the experiment, the animals were sacrificed by cervical dislocation and the samples were collected. 

##### Lipid Profile Analysis

Blood samples were taken after fasting for 15 h, and the serum was obtained by centrifugation at 2500 rpm for 10 min. Concentrations of total cholesterol (TC), triglycerides (TG), high-density lipoprotein (HDL), aspartate transaminase (AST), and alanine transaminase (ALT) were measured using commercial diagnostic kits, and the data were represented as mean mg/dL ± standard deviation. Low-density lipoprotein (LDL) concentrations were calculated using the Friedewald equation. Biochemical parameters were evaluated by a one-way ANOVA test using Statgraphics (Statgraphics Technologies, The Plains, VA, USA) followed by Tukey’s test.

##### Histological Analysis

A 5 mm thick section of liver tissue was cut and fixed in 40 g/L buffered formaldehyde and embedded in paraffin. The sections were stained with hematoxylin and eosin using a morphological semi-quantitative approach and graded as follows: steatosis: 0–3; inflammation: 0–3; ballooning: 0–2 [18]. The histological evaluation of the liver sections was performed by an experimental pathologist.

## 3. Results and Discussion

### 3.1. SEM Characterization

**Figure 1:**
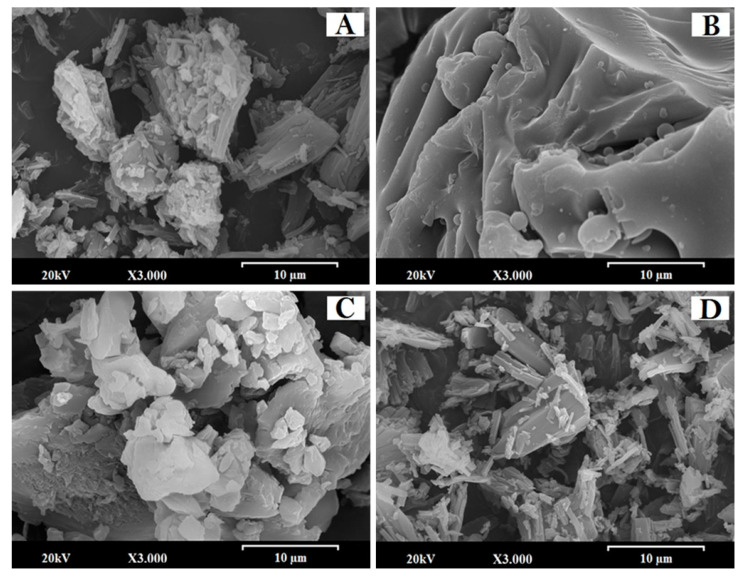
SEM micrographs of surface-modified ezetimibe (EZ) and atorvastatin calcium (ATV) formulations: (**A**) Solid dispersion (SD)-EZ (1:2.5), (**B**) micellar system (MS)-EZ (1:1), (**C**) physical mixture (PM)-ATV (1:1), and (**D**) SD-ATV (1:1). Photographs were taken at a magnification of 3000×.

The EZ solid dispersion showed small crystals of the active ingredient (2–5 µm) on the surface of large smooth particles of croscarmellose (Figure 1A). However, when Kolliphor RH40 was added to the EZ micellar system (Figure 1B), the SEM micrograph showed a surfactant film covering the small crystals of EZ on the large croscarmellose particles identified by energy dispersive X-ray (EDX) analysis. The presence of the surfactant film decreases the crystallinity of EZ and increases its wettability in aqueous media [11,19].

Figure 1C shows the surface of ATV physical mixture. The small lighter-colored ATV particles (between 1 and 5 µm) are easily identifiable on the surface of the croscarmellose carrier. The ATV solid dispersion showed a significant change in the morphology of the ATV (Figure 1D), with some small ATV particles (2–5 µm) detected with an energy dispersive X-ray (EDX) on the smooth surface of the croscarmellose carrier. Some particles of the active ingredient may not be included within the croscarmellose polymer complex, leading to the formation of small ATV particles on the surface of the solid dispersion. A similar process whereby the amount of small ATV crystals is increased has been described in nanocrystalline structures [20,21].

### 3.2. Differential Scanning Calorimetry (DSC)

**Figure 2:**
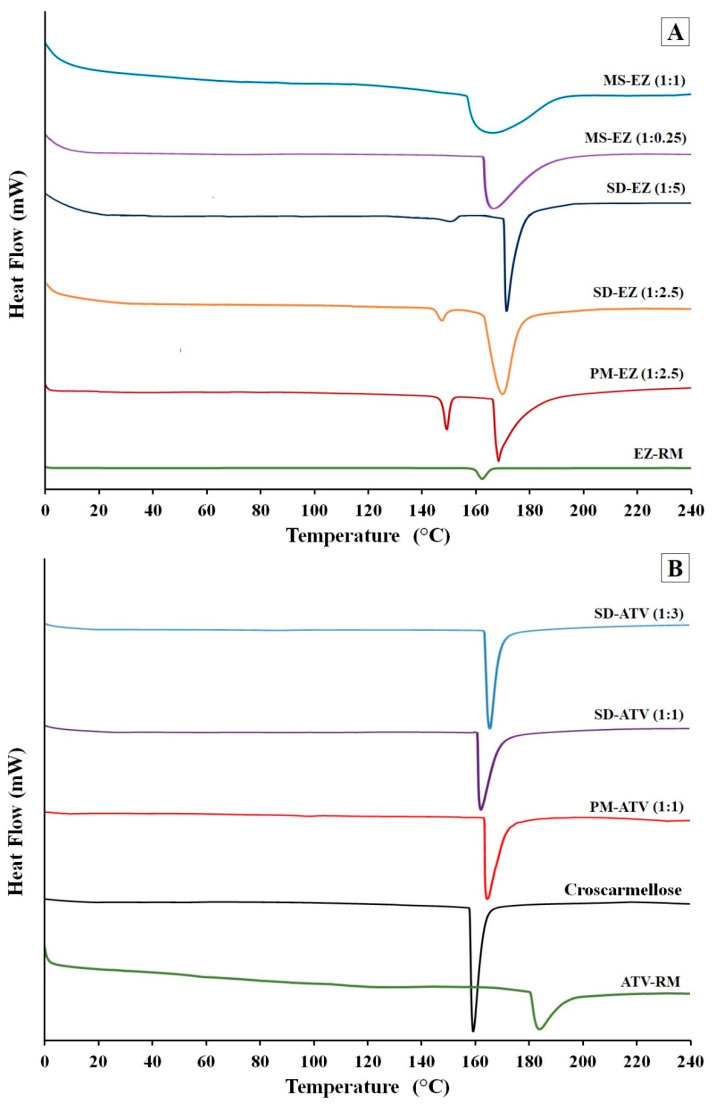
Differential scanning calorimetry (DSC) thermograms of ezetimibe (**A**): EZ raw material (EZ-RM), PM-EZ (1:2.5), SD-EZ (1:2.5), SD-EZ (1:5), MS-EZ (1:0.25), and MS-EZ (1:1). DSC thermograms of atorvastatin (**B**): ATV raw material (ATV-RM), croscarmellose, PM-ATV (1:1), SD-ATV (1:1), and SD-ATV (1:3).

The EZ-RM (Figure 2A) displayed an endothermic peak at 162.24 °C and an enthalpy value of 77.00 J/g, caused by a micronized form of EZ [7]. The hydrophilic croscarmellose used to prepare the ezetimibe formulations exhibited a sharp melting peak at 159.37 °C and an enthalpy value of 2542.84 J/g. A similar endothermic peak has previously been observed in other cellulose derivatives [22].

The physical mixture PM-EZ (1:2.5) showed a first endothermic peak at 150.34 °C (Figure 2A), corresponding to the interaction of the ezetimibe molecules with the croscarmellose chains. The second endothermic peak at 168.76 °C was related to the crystalline domain of the croscarmellose polymer chains. The lower melting temperature of the active ingredient compared to EZ-RM melting peak and the slight shift of croscarmellose temperature in PM-EZ (1:2.5) could be the result of a miscibility between the ezetimibe and croscarmellose in the physical mixture. 

The solid dispersions showed a first small broad endothermic peak of EZ at 149.63 °C for SD-EZ (1:2.5) and 151.83 °C for SD-EZ (1:5) in Figure 2A. The change of enthalpy analyzed in this endothermic peak was attributed to the miscibility of EZ with croscarmellose in the solid dispersions. This interaction EZ/croscarmellose decreased the crystallinity, showing an enthalpy reduction of EZ peak. A second melting point was observed in SD-EZ (1:2.5) at 171.67 °C, and in SD-EZ (1:5) at 169.46 °C, characteristic of croscarmellose polymer. The higher size of the peak was related to a greater amount of crystalline domain of croscarmellose chains present in this formulation as a result of the miscibility of the active ingredient and the polymer during the preparation of the solid dispersions. The increment of the croscarmellose polymer proportion in SD-EZ (1:5) presented a sharper peak of croscarmellose. Similar changes in the enthalpy and size of the peaks were observed in solid dispersions with cellulosic derivatives [22,23]. 

MS-EZ (1:0.25) and MS-EZ (1:1) in Figure 2A exhibited a single endothermic peak of croscarmellose. MS-EZ (1:0.25) showed this melting peak at 167.53 °C and MS-EZ (1:1) croscarmellose peak at 166.49 °C; this change of the croscarmellose melting temperature could be a result of the EZ/polymer interaction with the Kolliphor RH40 added in these formulations. In both micellar systems, the absence of the first endothermic peak characteristic of EZ was attributed to a decrease of EZ crystallinity with the addition of Kolliphor RH40. The higher ratio of surfactant in MS-EZ (1:1) increased the mobility of croscarmellose chains and the inclusion of the ezetimibe [19]. These surfactant ratios make it possible to improve the interaction between EZ particles and croscarmellose chains, obtaining amorphous forms of EZ in these formulations with low ratios of hydrophilic carriers [4,22]. 

The DSC thermograms in Figure 2B had an endothermic peak of ATV-RM at 184.94 °C with an enthalpy of 298.23 J/g, corresponding to a semi-crystalline form of ATV as observed in the SEM studies [15]. The PM-ATV (1:1) showed a single peak at 165.48 °C, indicating a miscibility between the atorvastatin and croscarmellose carrier. The physical mixture of ATV showed an endothermic transition at 98.58 °C attributed to a dehydration and recrystallization process.

SD-ATV (1:1) and SD-ATV (1:3) presented endothermic peaks at 163.02 and 166.52 °C, respectively (Figure 2B). The low temperatures of these endothermic peaks compared to ATV-RM are related to the strong ATV/croscarmellose interaction. Both solid dispersions showed a single peak as a result of the interaction between ATV particles and croscarmellose chains, presented at the same melting temperature point for both components. The sharper endothermic peak of SD-ATV (1:3) showed an increase in croscarmellose crystallinity (111.60%) due to a recrystallization of a higher proportion of the polymer. However, the smaller proportion of the croscarmellose in SD-ATV (1:1) produced a decrease in crystallinity (76.33%) compared to PM-ATV (1:1). The presence of a lower proportion of this carrier decreases the size of the peak as a result of the reduction in the crystalline domain of the croscarmellose [9,21,23].

### 3.3. Powder X-ray Diffraction (PXRD): Structure and Crystal Size Characterization

**Figure 3:**
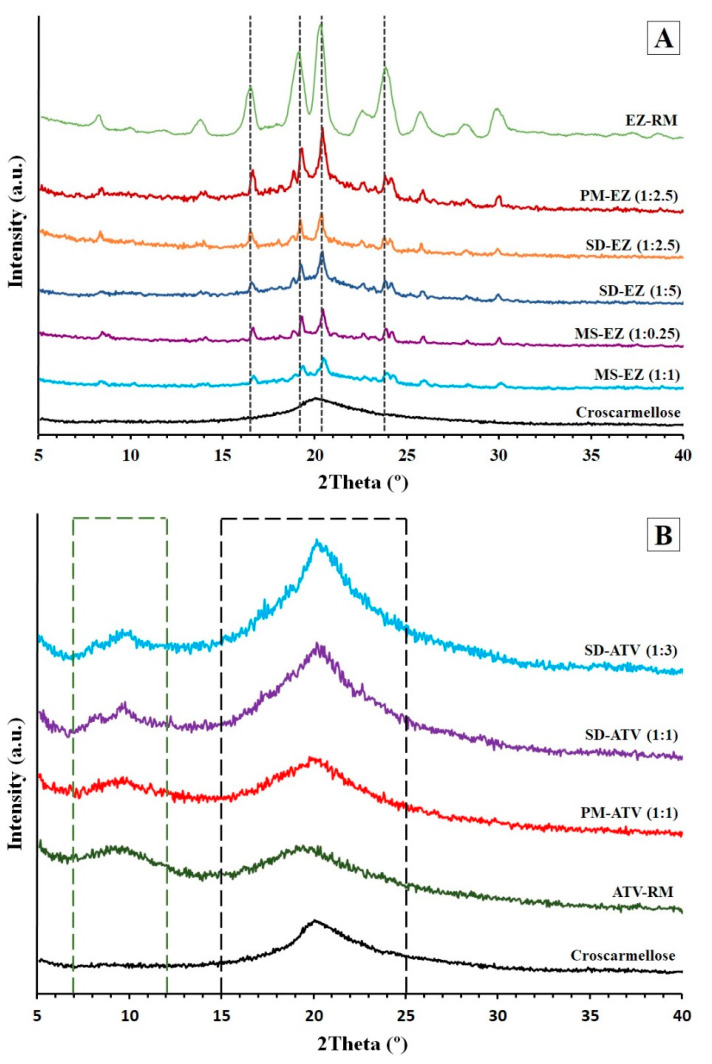
Powder X-ray diffraction (PXRD) patterns of ezetimibe (**A**): EZ raw material (EZ-RM), PM-EZ (1:2.5), SD-EZ (1:2.5), SD-EZ (1:5), MS-EZ (1:0.25), MS-EZ (1:1), and croscarmellose. PXRD diffraction patterns of atorvastatin (**B**): croscarmellose, ATV raw material (ATV-RM), PM-ATV (1:1), SD-ATV (1:1), and SD-ATV (1:3).

The X-ray diffraction patterns in Figure 3A show the EZ-RM with representative peaks at 16.44°, 19.13°, 20.37°, and 23.85° 2θ. This crystalline structure corresponds to the anhydrous form of ezetimibe [7].

The physical mixture PM-EZ (1:2.5) showed a semi-crystalline halo between 15 and 25° 2θ, where the most representative peaks of EZ can be seen to have a lower intensity compared to EZ-RM (Figure 3A). This semi-crystalline halo is due to the croscarmellose carrier, and to the lower intensity of PM-EZ (1:2.5) peaks to the dilution effect caused by this cellulose polymer [9].

The micellar system with low surfactant ratios MS-EZ (1:0.25) had similar intensity values to those observed in the solid dispersions (54.88%) for the ezetimibe diffraction peaks. However, the use of high amounts of surfactant in the MS-EZ (1:1) formulation produced a mostly amorphous structure of ezetimibe, with a low crystal size of 28.87% compared to PM-EZ (1:2.5). These results indicate that the presence of high Kolliphor RH40 ratios produced significant drug–surfactant interactions and increased the amorphous form of ezetimibe. Previous studies have shown that the micellar system is able to change the biodistribution of lipophilic drugs [6,24,25].

ATV-RM PXRD (Figure 3B) presented two different semi-crystalline halos with diffraction angles between 7 and 12°, and 15 and 25° 2θ, respectively, and were related to the atorvastatin amorphous form used in the development of new commercial formulations [26]. This amorphous form of atorvastatin derives from the morphology of ATV-RM observed in the SEM studies. PM-ATV (1:1) showed a first semi-crystalline halo between 7 and 12° 2θ, and a second broad halo between 15 and 25° 2θ, due to the inclusion of ATV-RM within the semi-crystalline halo of the croscarmellose (Figure 3B).

The SD-ATV (1:1) and SD-ATV (1:3) solid dispersions showed a semi-crystalline halo of atorvastatin between 7 and 12° 2θ, similar to the PM-ATV (1:1) formulation, and a high intensity halo between 15 and 25° 2θ (Figure 3B). The higher intensity of this second halo could be a result of the recrystallization of croscarmellose during the elaboration process of the solid dispersions. The evaporation of the solvent during drying process could increase the crystalline domain of the cellulosic polymer. The higher proportion of croscarmellose for SD-ATV (1:3) could explain the greater intensity of the halo between 15 and 25° 2θ, compared to SD-ATV (1:1). The smaller proportion of croscarmellose improves the ATV–croscarmellose interactions and reduces the crystallinity of ATV. A similar molecular interaction of this drug with other cellulose polymers has been observed previously [25]. 

### 3.4. In Vitro Drug Release

#### 3.4.1. Dissolution Test under Sink Conditions

**Figure 4:**
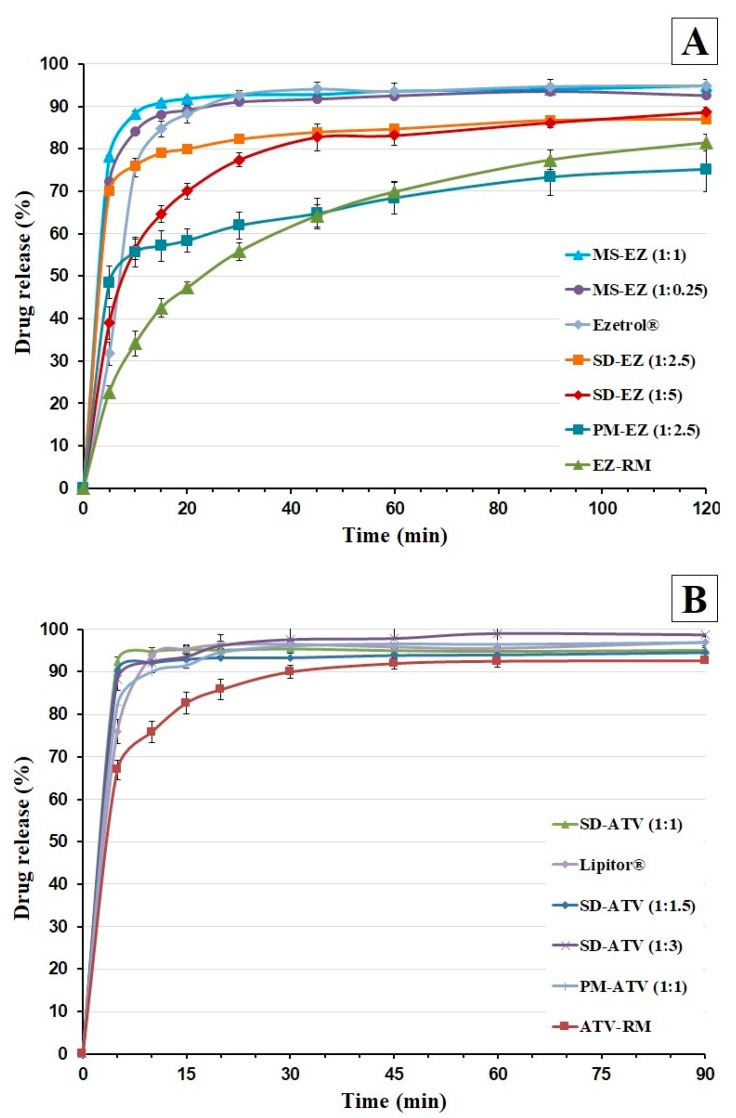
Dissolution profiles under sink conditions of ezetimibe (**A**): EZ raw material (EZ-RM), PM-EZ (1:2.5), SD-EZ (1:2.5), SD-EZ (1:5), Ezetrol, MS-EZ (1:0.25), and MS-EZ (1:1). Dissolution profiles of atorvastatin (**B**): ATV raw material (ATV-RM), PM-ATV (1:1), Lipitor, SD-ATV (1:1), SD-ATV (1:1.5), and SD-ATV (1:3).

EZ-RM (Figure 4A) had a slow dissolution profile with percentages of 34.15 ± 3.02% at 10 min, due to the formation of agglomerates during the recrystallization process [3,7]. However, PM-EZ (1:2.5) showed a significant 1.63-fold increase (*p* < 0.05) at 10 min compared to EZ-RM (Figure 4A). The improvement in the wettability of the croscarmellose carrier avoids the aggregation of hydrophobic drugs [9,21,25].

The micellar systems MS-EZ (1:0.25) and MS-EZ (1:1) showed significant increases of 2.46- and 2.59-fold (*p* < 0.05) at 10 min compared to EZ-RM (Figure 4A). Different studies indicated that surfactants such as Kolliphor RH40 or Tween 80 are especially suitable for the formation of micelles and increase the solubility of poorly solubility drugs [27,28]. The commercial tablet Ezetrol showed a release percentage of 75.52 ± 2.13% at 10 min. The presence of the surfactant SDS in the Ezetrol tablets improved the dissolution profile and exerted an inhibitory effect on P-glycoprotein [21,29]. Various studies indicate that surfactants such as Kolliphor RH40 or Tween 80 produce micelles that are especially suitable for increasing the solubility of low-soluble drugs [5,25].

Figure 4B shows the dissolution profiles of the atorvastatin formulations. ATV-RM had a fast dissolution profile with percentages of 66.89 ± 1.65% at 5 min and more than 75% at 10 min. The improvement in dissolution profiles for ATV-RM was due to the amorphous form of ATV-RM in our study compared to the crystalline form of ATV used in other articles [15]. The dissolution profiles of the PM-ATV (1:1) showed a significant 1.22-fold increase (*p* < 0.05) at 5 min compared to ATV-RM (Figure 4B). This improvement is explained by the swelling and water uptake of the hydrophilic carrier [7,9]. These improvements of dissolution profile could by related to the wettability increment of the amorphous form of ATV used in the formulation of commercial tablets [26].

The commercial tablet Lipitor had lower dissolution percentages at 5 min (75.97 ± 2.81%) compared to PM-ATV (1:1), due to the compression process of the commercial tablets. The improvement of Lipitor at 10 min may be related with the use of a surfactant and the greater surface area and wettability in the dissolution medium [15].

The solid dispersions SD-ATV (1:1), SD-ATV (1:1.5), and SD-ATV (1:3) showed percentages of 92.44 ± 0.22%, 90.12 ± 0.44%, and 88.14 ± 0.95% at 5 min, respectively (Figure 4B). However, there were no improvements in the dissolution profile of SD-ATV (1:1.5 and 1:3) with high proportions of croscarmellose compared to SD-ATV (1:1). These changes in lipophilic drugs such as voriconazole have been studied previously [10]. The delayed dissolution profiles for SD-ATV (1:1.5) and SD-ATV (1:3) were related to a partial recrystallization of the croscarmellose. Similar delays in the dissolution profiles have been observed with high ratios of different hydrophilic polymers [25]. The use of low carrier ratios in solid dispersions has shown to be adequate in previous studies with hydrophilic polymers [30,31].

This SD-ATV (1:1) solid dispersion increased the solubility in the diffusion layer through the rapid dispersion of the atorvastatin particles in the dissolution medium without the use of surfactants. Various hydrophilic polymers have been studied to increase the dissolution of poorly soluble drugs and avoid the aggregation of its particles [14,15].

#### 3.4.2. Dissolution Test in Biorelevant Media under Non-Sink Conditions

**Figure 5:**
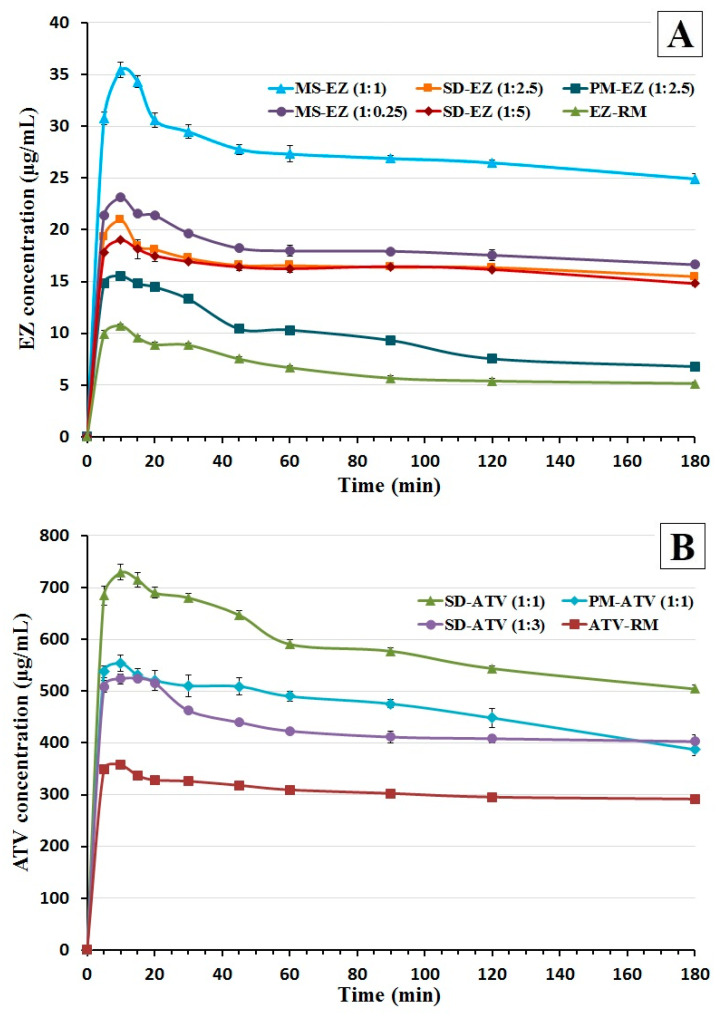
Dissolution profiles under non-sink conditions in fasted-state simulated intestinal fluid (FaSSIF) media of ezetimibe (**A**): EZ raw material (EZ-RM), PM-EZ (1:2.5), SD-EZ (1:2.5), SD-EZ (1:5), MS-EZ (1:0.25), and MS-EZ (1:1). Dissolution profiles of atorvastatin (**B**): ATV raw material (ATV-RM), PM-ATV (1:1), SD-ATV (1:1), and SD-ATV (1:3).

The dissolution rate of EZ-RM in supersaturation conditions (Figure 5A) showed a poor dissolution (10.73 ± 0.19 µg/mL at 10 min) and a fast precipitation in FaSSIF biorelevant media at pH 6.5. However, the precipitation of EZ was delayed in physical mixture PM-EZ (1:2.5). Croscarmellose polymer added in this formulation prevents the rapid precipitation process of the active ingredient in biorelevant media [16].

The SD-EZ (1:2.5) showed a significant 1.96-fold increase (*p* < 0.05) at 10 min in FaSSIF media compared to EZ-RM (Figure 5A). The supersaturation in this formulation is thermodynamically more stable with lower precipitation tendencies as a result of the solid dispersion with croscarmellose. Different polymers such as croscarmellose or PVP maintain supersaturation solution of the active ingredient [24,32]. However high proportions of hydrophilic polymer added in SD-EZ (1:5) did not increase the EZ concentration. These results of SD-EZ (1:5) are similar to those obtained in the dissolution test under sink conditions and are probably caused by an extensive swelling of the cellulosic polymer [33].

Micellar systems with different proportions of surfactant showed the highest EZ concentrations in FaSSIF media under non-sink conditions (Figure 5A). MS-EZ (1:0.25) presented a slight increase in comparison with EZ solid dispersions, with similar supersaturation profile to SD-EZ (1:2.5). However, the micellar system MS-EZ (1:1) showed a significant 3.30-fold increase (*p* < 0.05) at 10 min compared to EZ-RM. The increment of drug release for the first 10 min and high supersaturation concentration of EZ during the 3 h of dissolution test in biorelevant media allowed us to consider the use of MS-EZ (1:1) for the in vivo studies. Previous articles have studied the supersaturation of EZ concentrations with surfactant, showing similar result with the elaboration of ternary solid dispersions [33] and delaying precipitation process of the active ingredient [24,32].

The dissolution of the ATV amorphous form under non-sink conditions in biorelevant media showed a concentration of 357.13 ± 5.20 µg/mL for ATV-RM at 10 min (Figure 5B). The elaboration of a physical mixture PM-ATV (1:1) presented a significant 1.55-fold increase (*p* < 0.05) at 10 min compared to ATV-RM. The increment of ATV dissolution and supersaturation concentration for this study may be result of the croscarmellose hydrophilic carrier added [34].

The elaboration of a solid dispersion with high proportions of croscarmellose SD-ATV (1:3) showed similar concentrations compared to physical mixture. However, the solid dispersion SD-ATV (1:1) presented a significant 2.04-fold increase (*p* < 0.05) compared to ATV-RM. In comparison with SD-ATV (1:3), the lower proportion of croscarmellose polymer in SD-ATV (1:1) showed a significant 1.39-fold increase in the dissolution test under non-sink conditions (Figure 5B). The lower proportion of polymer used in the preparation of the solid dispersion SD-ATV (1:1) can reduce de-recrystallization of the polymer and the active ingredient compared with SD-ATV (1:3), increasing the dissolution rate of ATV and its release in physiological media [34,35]. Previous studies of ATV formulations with hydrophilic polymer have shown improvements in the solubility of this drug [27,28].

### 3.5. Effects of the Treatments on the Lipid Profile

The study was conducted with type 2 diabetic GK rats fed a high-fat diet and treated with EZ/ATV-RM and multiparticulate systems containing MS-EZ (1:1) and SD-ATV (1:1) at two different doses of EZ and ATV (MPS-I and MPS-II). GK rats fed a high-fat diet (HFD group) exhibited a significant (*p* < 0.05) increase in TC (53.65%), TG (72.69%), and LDL (145.11%), and a significant decrease (*p* < 0.05) in HDL (43.95%) compared with the control group (Table 1). The use of diabetic rats causes a substantial reduction in the hepatic LDL receptor, increasing the highest levels of cholesterol and LDL [1,2].

Treatment with EZ/ATV-RM at high doses (3/10 mg/kg) led to a significant reduction (*p* < 0.05) in TC (21.31%), TG (21.02%), and LDL (32.73%) compared to the HFD group (Table 1). These doses of EZ/ATV were suitable for partially reducing lipid profiles [6].

After treatment, the lipid values were significantly lower (*p* < 0.05) in the MPS-I (3/10 mg/kg EZ/ATV), with decreases of 25.58%, 35.97%, and 42.12% for TC, TG, and LDL compared to the EZ/ATV-RM group. These improvements with the administration of the multiparticulate system could be related to the inhibitory effect of Kolliphor RH40 on the P-glycoprotein efflux. The surfactant can inhibit the activity of P-glycoprotein and increase the accumulation of substrate drugs such as ezetimibe and statins [29,36,37]. The MPS-II (2/6.7 mg/kg EZ/ATV) group showed reductions (*p* < 0.05) of 25.37% for TC, 34.83% for TG, and 44.13% for LDL levels compared to the EZ/ATV-RM group (Table 1). These similar values in MPS-I and MPS-II pointed to the possibility of reducing cholesterol and lipid levels using multiparticulate systems with lower doses of EZ and ATV (MPS-II). Low doses may play a key role in mitigating the adverse effects of atorvastatin [6]. Similar improvements in the hypolipidemic effect in diabetic rats have been attributed to an antioxidant effect [12,38].

Elevated liver steatosis has also been studied in this type of diabetic rat [2]. The ALT and AST levels (Table 1) for the HFD group point to oxidative stress and are related to liver damage [12]. After treatment with EZ/ATV-RM, ALT and AST levels decreased by 13.15% and 25.18%, respectively, compared to the HFD group. Previous work has shown a clear relationship between the reduction in ALT and AST levels and the anti-inflammatory and antioxidant action of various substances [12,18,38]. The ALT and AST reductions obtained with the fast-dissolving formulations MPS-I (8.35% and 7.33%) and MPS-II (9.21% and 17.39%) compared to EZ/ATV-RM group indicated an improvement in hepatic steatosis. The surfactant Kolliphor RH40 may inhibit the efflux transporter (P-glycoprotein) and improve the elimination of fatty acids from liver cells [37,39].

### 3.6. Histopathological Study

**Figure 6:**
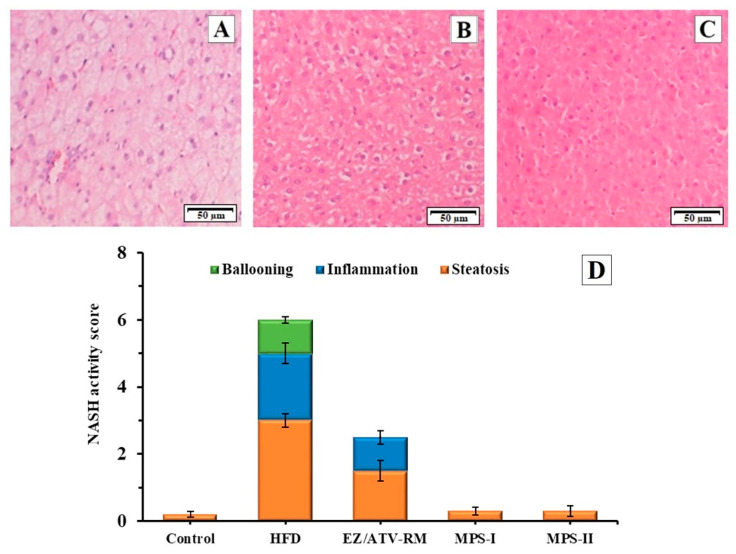
Photomicrography (hematoxylin and eosin, 20X) of liver tissues in GK (diabetic) rats after 8 weeks of treatment. (**A**) HFD group; (**B**) ezetimibe/atorvastatin raw material (EZ/ATV-RM); (**C**) multiparticulate system MPS-II. (**D**) Histological evaluation of the liver sections. Mean of NASH score and standard error (*n* = 6) of the different GK rat groups: Control GK group (Control), high-fat diet group (HFD), ezetimibe/atorvastatin raw material (EZ/ATV-RM), multiparticulate systems MPS-I and MPS-II.

The histopathological examination of the livers of GK groups is shown in Figure 6. The histological examination of normal liver tissue in the control group revealed no inflammation, ballooning, or fibrosis. Only some animals presented a low level of steatosis. The HFD group (Figure 6A) showed a severe degree of steatosis, with intracellular vacuolation in hepatocytes and movement of the nucleus to the peripheral area. The histopathological studies in this group also revealed a small quantity of infiltrating inflammatory cells and a low level of ballooning degeneration. These results indicated non-alcoholic fatty liver disease (NAFLD) with high NASH score (Figure 6D). Similar NASH scores were observed in different studies on diabetic rats fed a high-fat diet [18]. These degenerations in liver tissue are related to the high lipid levels and the oxidative stress observed in other studies with diabetic animals fed a high-fat diet [1,12].

The EZ/ATV-RM group at doses of 3/10 mg/kg showed a sharp decrease in the NASH score compared to the HFD group (Figure 6D). This group had a moderate steatosis and inflammatory cell infiltration, as well as an absence of ballooning degeneration (Figure 6B). Similar values of inflammation and ballooning with high steatosis values have been described in previous studies with different treatments of ezetimibe or atorvastatin in insulin-resistant obese rats [1]. These results match the changes in the visual appearance of the liver samples (Figure S1).

A significant improvement was observed in the histopathological analyses after treatment with MPS-I and MPS-II (Figure 6C) compared to the HFD group and EZ/ATV-RM group. Histological micrographs of both multiparticulate systems showed similar NASH values, with a significant recovery for hepatic steatosis and an absence of inflammatory cells and ballooning degeneration. The NASH score for both the MPS-I and MPS-II groups showed no significant differences compared to the control group (Figure 6D). These results are consistent with the recovery observed in the visual appearance of the livers (Figure S1). Other treatments with antioxidants and anti-inflammatory substances showed significant improvements in the NASH score but with no reductions in steatosis [18]. Previous studies with other multiparticulate systems have related the hepatic effect with low steatosis values in liver cells [6,20,38]. These results allow us to consider the use of MPS-II with lower doses of EZ/ATV (2/6.7 mg/kg) to reduce hepatic steatosis in diabetic rats.

## 4. Conclusions

In this study, micellar systems and solid dispersions were developed to increase the dissolution rate of ezetimibe and atorvastatin. The formulations were characterized with the SEM, PXRD, and DSC techniques. In the characterization of MS-EZ formulations, the surfactant/drug and surfactant–polymer interactions reduce the crystallinity of the ezetimibe and croscarmellose chains. MS-EZ (1:1) was selected for its fast dissolution profile. The solid dispersion of atorvastatin with low proportions of croscarmellose SD-ATV (1:1) showed drug–polymer interactions with a reduction in the crystallinity of atorvastatin and croscarmellose (observed in the PXRD and DSC studies). These results were related to its rapid dissolution rate.

The serum lipid levels and transaminase values for the MPS-I and MPS-II groups improved significantly compared to the HFD group in GK diabetic rats. The improvement in the NASH score for MPS-II with low doses of EZ and ATV (2/6.7 mg/kg) compared to EZ/ATV-RM with high doses (3/10 mg/kg EZ/ATV) indicated that the multiparticulate system with lower doses of the active ingredients and Kolliphor RH40 enhanced the reduction of lipid fats in liver cells.

## Figures and Tables

**Table 1:** Serum levels of total cholesterol (TC), triglycerides (TGs), low-density lipoproteins (LDLs), high-density lipoproteins (HDLs), aspartate transaminase (AST), and alanine transaminase (ALT) after 8 weeks of treatment. Mean and standard error (*n* = 6) of the following Goto-Kakizaki (GK) rat groups: control group (Control), high-fat diet group (HFD), ezetimibe/atorvastatin raw material (EZ/ATV-RM), multiparticulate systems MPS-I and MPS-II.

	Control	HFD	EZ/ATV-RM	MPS-I	MPS-II
**TC** (mmol/L)	117.80 ± 8.17	181.00 ± 21.56	142.43 ± 10.20 ӿ	106.00 ± 11.15 ӿ #	106.29 ± 8.24 ӿ #
**TG** (mmol/L)	98.25 ± 16.88	169.67 ± 11.38	134.00 ± 15.71 ӿ	85.80 ± 18.58 ӿ #	87.33 ± 10.07 ӿ #
**LDL** (mmol/L)	48.55 ± 5.14	119.27 ± 7.94	80.23 ± 7.22 ӿ	46.44 ± 6.13 ӿ #	44.82 ± 5.85 ӿ #
**HDL** (mmol/L)	49.60 ± 4.27	27.80 ± 4.97	35.40 ± 3.21	42.40 ± 5.13 ӿ	44.00 ± 4.06 ӿ #
**ALT** (U/L)	74.40 ± 8.02	80.40 ± 9.13	69.83 ± 7.86	64.00 ± 7.35 ӿ	63.40 ± 7.96 ӿ
**AST** (U/L)	180.00 ± 11.05	205.17 ± 25.42	153.50 ± 19.82 ӿ	142.25 ± 14.80 ӿ	126.80 ± 17.57 ӿ

(ӿ) significant difference (*p* < 0.05) compared with HFD group; (#) significant difference (*p* < 0.05) of MPS-I and MPS-II groups compared with EZ/ATV-RM group.

## Data Availability

Not applicable.

## Supplementary Materials

The following are available online at https://www.mdpi.com/1999-4923/13/3/421/s1, Figure S1. Photomicrography (hematoxylin-eosin, 20X) and visual appearance of the liver tissues in GK (diabetic) rats after 8 weeks of treatment.
