# Supplementary material for: Multiparticulate Systems of Ezetimibe Micellar System and Atorvastatin Solid Dispersion Efficacy of Low-Dose Ezetimibe/Atorvastatin on High-Fat Diet-Induced Hyperlipidemia and Hepatic Steatosis in Diabetic Rats
Source: Pharmaceutics. 2021 Mar 20;13(3):421. doi: 10.3390/pharmaceutics13030421 (PMC8004026; doi:10.3390/pharmaceutics13030421)
Supplement: Supplementary file 1 [file pharmaceutics-13-00421-s001.pdf]

pharmaceutics

Supplementary Materials: Multiparticulate Systems of Ezetimibe Micellar System and Atorvastatin Solid Dispersion Efficacy of Low-Dose Ezetimibe/Atorvastatin on High-Fat Diet-Induced Hyperlipidemia and Hepatic Steatosis in Diabetic Rats

Carlos Torrado-Salmerón [1], Víctor Guarnizo-Herrero [1], Joana Henriques [2], Raquel Seiça [2], Cristina M. Sena [2] and Santiago Torrado-Santiago [1,3,*]

Photomicrography and visual appearance of the liver samples.

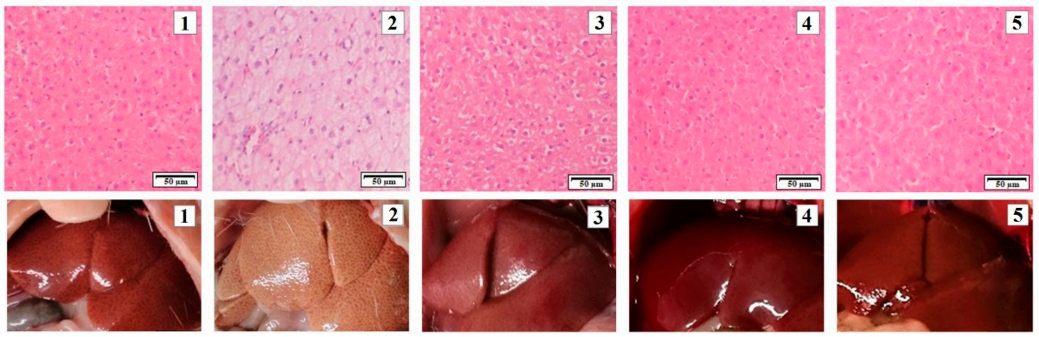

Figure S1. Photomicrography (hematoxylin-eosin, 20X) and visual appearance of the liver tissues in GK (diabetic) rats after 8 weeks of treatment. (1) Control group (Control), (2) HFD group, (3) ezetimibe/atorvastatin raw material (EZ/ATV-RM), (4) multiparticulate system MPS-I and (5) multiparticulate system MPS-II.
